# Adaptation and analysis of the Hungarian version of the Snaith-Hamilton Pleasure Scale

**DOI:** 10.1192/j.eurpsy.2024.1114

**Published:** 2024-08-27

**Authors:** V. Koumou, B. K. Nagy, B. Kabella, A. Feher, L. Pogany, J. Lazary

**Affiliations:** ^1^National Institute of Mental Health, Neurology and Neurosurgery; ^2^Semmelweis University, Budapest, Hungary

## Abstract

**Introduction:**

Anhedonia (loss of ability of experience pleasure) is a transdiagnostic symptom which is presented as a prominent complain in several psychiatric disorders, such as depressive disorders, schizophrenia, bipolar disorder, addictive disorders, certain personality disorders etc. Specific instruments for assessment of anhedonia have been published in the international literature but their Hungarian versions are not available so far, however, the prevalence of affective disorders and suicide are also high in Hungary. The Snaith-Hamilton Pleasure Scale (SHAPS) is an instrument developed in 1995 (Snaith et al. Br J Psychiatry1995;167:99-103) which purposley has been constructed with items that can be easily translated into other languages.

**Objectives:**

The aim of our study was to translate the 14 items into Hungarian and analyse its reability and sensitivity in a Hungarian sample consists of patinets and control persons. Further aim was to explore the differences of anhedonia profiles among diagnostic categories and subgroup of major disorders.

**Methods:**

We recruited 170 subjects (101 controls and 59 patients; 78 men and 82 women; mean age=37,9±6,1y) into our study. Among the patients there were 27 subjects with major depressive disorer (MDD), 10 subjects with bipolar disorder (BD), 9 patients with schizophrenia (SCZ), 6 patients with addictive disorder (AD) and 7 patients with anxiety disorder (ANX)±. We created two major subgorups from the dfferent diagnostic categories: affective and psychotic subgroups to compare the anhedonic profiles. Differences of mean values between case and control, men and women and subgroups were analysed by t-tests and diganostic categories by ANOVA tests performing in SPSS 20.0 software.

**Results:**

Among the MDD, the BD, the SCZ, the AD and the ANX groups, patients with MDD produced the highest score (6.9±3.5; 3.9±2.4; 5.9±3.9; 2.8±2.7;2.3±1.8, respectively), while controls prohibited 1.6±1.3. The case group scored significantly higher on the SHAPS than the control group (5.3± 3.6 vs. 1.6±1.3; *p*=0.0001). The means of SHAPS did not differ significantly between the affective subgroup and the psychotic subgroup (6.0±3.7 vs. 4.8±3.2; *p*=0.24). Among the subgroup of women, the age was significantly associated with the SHAPS score (*p*=0,04), however, this association has been not detected in men.

**Conclusions:**

The Hungarian version of the SHAPS detected marked difference between cases and controls with good reliability and sensitivity. The instrument can be useful in daily clinical routin becuase subjects could fill it easily and quickly. In case of patients with pronounced anhedonia, treatments with spcifically targeting anhedonia can be preferred (e.g. rTMS as it was demonstrated in our earlier publications, see Lazary et al. Sci Rep 2021,11:8867; Elemery et al. Front Psychiatry 2022, 13:806731). This study was supported by the grant EFOP 5.6.2.

**Disclosure of Interest:**

None Declared

